# Silymarin effect on amyloid-β plaque accumulation and gene expression of APP in an Alzheimer’s disease rat model

**DOI:** 10.1186/2008-2231-22-24

**Published:** 2014-01-24

**Authors:** Parichehreh Yaghmaei, Katia Azarfar, Mehrooz Dezfulian, Azadeh Ebrahim-Habibi

**Affiliations:** 1 Department of Biology, Science and Research Branch, Islamic Azad University, Tehran, Iran; 2 Department of Microbiology, College of Science, Islamic Azad University, Karaj Branch, Karaj, Iran; 3 Biosensor Research Center, Endocrinology and Metabolism Molecular-Cellular Sciences Institute, Tehran University of Medical Sciences, Tehran, Iran; 4 Endocrinology and Metabolism Research Center, Endocrinology and Metabolism Clinical Sciences Institute, Tehran University of Medical Sciences, North Kargar Avenue, 1411413137 Tehran, Iran

**Keywords:** Silymarin, RT-PCR, Passive avoidance learning, Alzheimer’s disease, Beta amyloid

## Abstract

**Background:**

The deposition of amyloid peptides is associated with Alzheimer’s disease (AD). These amyloid peptides are derived from the amyloid protein precursor (APP). Silymarin, a standardized extract of milk thistle, which is currently used in liver diseases, may be effective in the inhibition of amyloid formation. However, its effect has not been assessed on APP expression.

**Results:**

In this study, first, the effect of silymarin was examined on the passive avoidance learning in a rat model of AD. This model was induced by the intracerebroventricular injection of Aβ peptide (Aβ_1–42_) in Wistar rats. Rats were treated with 70 and 140 mg/kgof the extract, once a day, for 4 weeks. Memory function that was evaluated in a shuttle-cage test, showed improvement upon administration of this extract. Brain amyloid plaques had also decreased upon administration of the extract. Furthermore, APP gene expression was compared in treated and untreated groups. The result showed that silymarin was able to suppress APP expression.

**Conclusion:**

Our results are in accordance with the *in vitro* tests concerning the positive antiamyloidogenic property of the main component of silymarin, namely silibinin. We suggest that the beneficial effect of sylimarin in the AD model is related to its capacity to disaggregate amyloid plaques and to suppress APP expression. Considering the limited side effects of silymarin, this compound could be of use in AD therapy.

## Background

Alzheimer’s disease (AD) is a progressive neurodegenerative disorder which has been characterized by the existence of extraneuronal aggregates of amyloid β (Aβ) peptide as well as intraneuronal deposits of hyperphosphorylated tau [1]. It is usually assumed that Aβ (abeta) aggregation in the brain is the starting trigger of a pathological cascade which ultimately leads to synaptic dysfunction and loss, neuronal death, and eventually cognitive dysfunction [2]; the process includes also oxidative stress and inflammatory response, which play their roles in neuronal dysfunction [3,4]. It is suggested that the reactive microglia and astrocytes that surround the Aβ plaques in the AD brain may release reactive oxygen species and proinflammatory molecules [5].

Various isoforms of Aβ peptide with lengths varying from 14 to 42 aminoacids have been characterized which are derivated from “APP”, the amyloid precursor protein (APP) [6,7]. However, it is generally accepted that the main dominant forms of Aβ (40 and 42 aminoacids) are the toxic species involved in AD pathophysiology, as for example, it has been shown that the presence of Aβ 42 in cerebrospinal fluid could be a reliable predictor of AD progression [8].

The intracerebroventricular administration of Aβ peptide into rodent brain has been used as a mean to simulate AD disease, since this injection could induce histological and biochemical changes as well as oxidative damage and inflammatory responses which result into memory deficits [9,10]. With this animal model, *in vivo* studies could be performed to test potential new candidates for AD therapy.

The flavonoid silibinin (or sylibin) [(2*R*,3*R*)-3,5,7-trihydroxy-2-[(2*R*,3*R*)-3-(4-hydroxy-3-methoxyphenyl)-2-(hydroxy-methyl)-2,3-dihydrobenzo[*b*][1,4]doxin-6-yl]chroman-4-one] is the main compound of the herb milk thistle (*Silybum marianum*) extract (silymarin) [11]. This compound possess anti-inflammatory and antioxidative effects [12]. It has also been reported that silymarin has protective effects against ethanol-induced brain injury [13], and neurotoxicity induced by lipopolysaccharide (LPS) [14]. In this study, we investigated the effect of silymarin on the memory impairment induced by Aβ1-42 injection in rats. We also examined its effect on changes in APP gene expression in the rats’ brain.

## Materials and methods

### Animals

Male Wistar rats weighing 250 ±300 g were housed at six per cage (42?×?26 cm), 23?±?0.5°C, under a 12/12-h light/dark cycle (lights on from 8:00 AM to 8:00 PM). Animals had free access to standard pellet food and water. Behavioral experiments were carried out in a sound-attenuated room, to which the rats were habituated for at least 1 hour. All experiments were performed in accordance with to the international guidelines set out in the *Guide for the Care and Use of Laboratory Animals* (Institute of Laboratory Animal Resources, 1996) and approved by the Research and Ethics Committee of Science and Research Branch, Azad University.

### Inducing Alzheimer’s disease in animals and used compound

Aβ1-42 (Sigma, St Louis, MO, USA), was dissolved in PBS and incubated at 37°C for 7 days, after which bilateral surgery and injection of Aβ1-42 peptide was done in rats hippocampus with the help of a stereotaxic apparatus (SR-6 N Narishig, Japan) [15]. Ketamine and xylazin (Alfasan,Woerden-Holland) were used to anesthetize the rats. Injection was performed slowly into the CA1 region of the hippocampus at both sides of the brain as mentioned in the Paxinos atlas [16].

Silymarin tablets, containing ethyl acetate extracted Silymarin, were purchased from Goldaru Pharmaceutical Laboratory, (Isfahan, Iran) and suspended in a distilled water solution. Treatment group was administered two different dosages of the compound (70 and 140 mg/kg/day P.O.) for 4 weeks after the injection with Aβ*1–42*. A volume of 0.1 ml/10 g body weight was used.

Animals were divided into four groups with n **=**?6: the control group did not undergo surgery, the sham group underwent surgery (AD inducing) and ws given distilled water 7 days after surgery, the experimental groups Exp1 and Exp2 received 70 and 140 mg/kg/day of the compound respectively (7 days after surgery).

### Testing the treatment efficacy

a. Passive avoidance test

A shuttle-cage consisting of two compartments of equal size (26?*×*?26 cm) separated by a sliding door (8?*×*?8 cm) was used. The shock compartment was dark in contrast to the starting compartment. Each experiment started with a pre-training trial, where the rat was placed in the starting compartment for 5 seconds, after which the sliding door was raised, and the rat was allowed to stay in the dark compartment for 10 seconds. The rat was then put back in its cage and stayed there for 30 minutes after which it was again put into the shuttle box, and this time, after entering the dark compartment, a footshock (50 Hz, 1 mA, and 5 s) was delivered. The rat was then put back into cage and stayed there for 120 seconds. When put back in the shuttle box, if a latency (in the order of 120 seconds) is observed before entering the dark compartment, successful acquisition of passive avoidance is recorded. A similar procedure was used 24 hours after training sessions to make a retention test for evaluating long-term memory. Higher or lower latencies are taken as indicative of increase or decrease in memory retention [17].

b. Histological studies of brain tissue

At the end of experiment animals were decapitated under anesthesia and their brain removed for histological assessments. First, the brains were fixed in 10% formalin and later processed for embedding with paraffin, after which serial sections in 6 mm of thickness were prepared. For staining of hippocampus cells, Thioflavin-S method which is detected by fluorescent microscopy was used [18].

c. Assessing amyloid precursor protein (APP) expression

Semi-quantitative RT-PCR was performed to assess APP expression. RNA was extracted from the homogenized brain tissue of the rats by use of RNX plus kit (CinnaGen, Iran). Isolated RNA was reverse-transcribed using the following gene-specific primers: 5′-GGA TGC GGA GTT CGG ACA TG -3′ (forward) and 5′-GTT CTG CAT CTG CTC AAA G -3′(reverse). GAPDH was used as housekeeping gene as a control [5′- GACATGCCGCCTGGAGAAAC -3 ' (forward) and 5′- AGCCCAGGATGCCCTTTAGT -3′ (reverse)]. The difference in threshold cycles between APP gene and GAPDH gives the standardized expression level.PCR were performed using the One-Step SYBR PrimeScript RT-PCR kit (Cinagen,Tehran-Iran). The reaction profile consisted of a first round at 95°C for 3 min and then 40 cycles of denaturation at 95°C for 30 s, annealing at 57°C for 30 s, and extension at 72°C for30 s, with a final extension reaction carried out at 72°C for 10 min. The RT-PCR products were loaded onto agarose gels and the resulting bands from electrophoresis were photographed with a UV transillumnator.

### Statistical analyses

The results are expressed as the mean?±?S.E.M. Statistical significance was determined with the one-way ANOVA followed by Tukey’s multiple comparisons test. A Pearson correlation analysis was performed to elucidate the relationships. *p* < 0.05 was taken as a significant level of difference.

## Results

### Passive avoidance test

**Figure 1:**
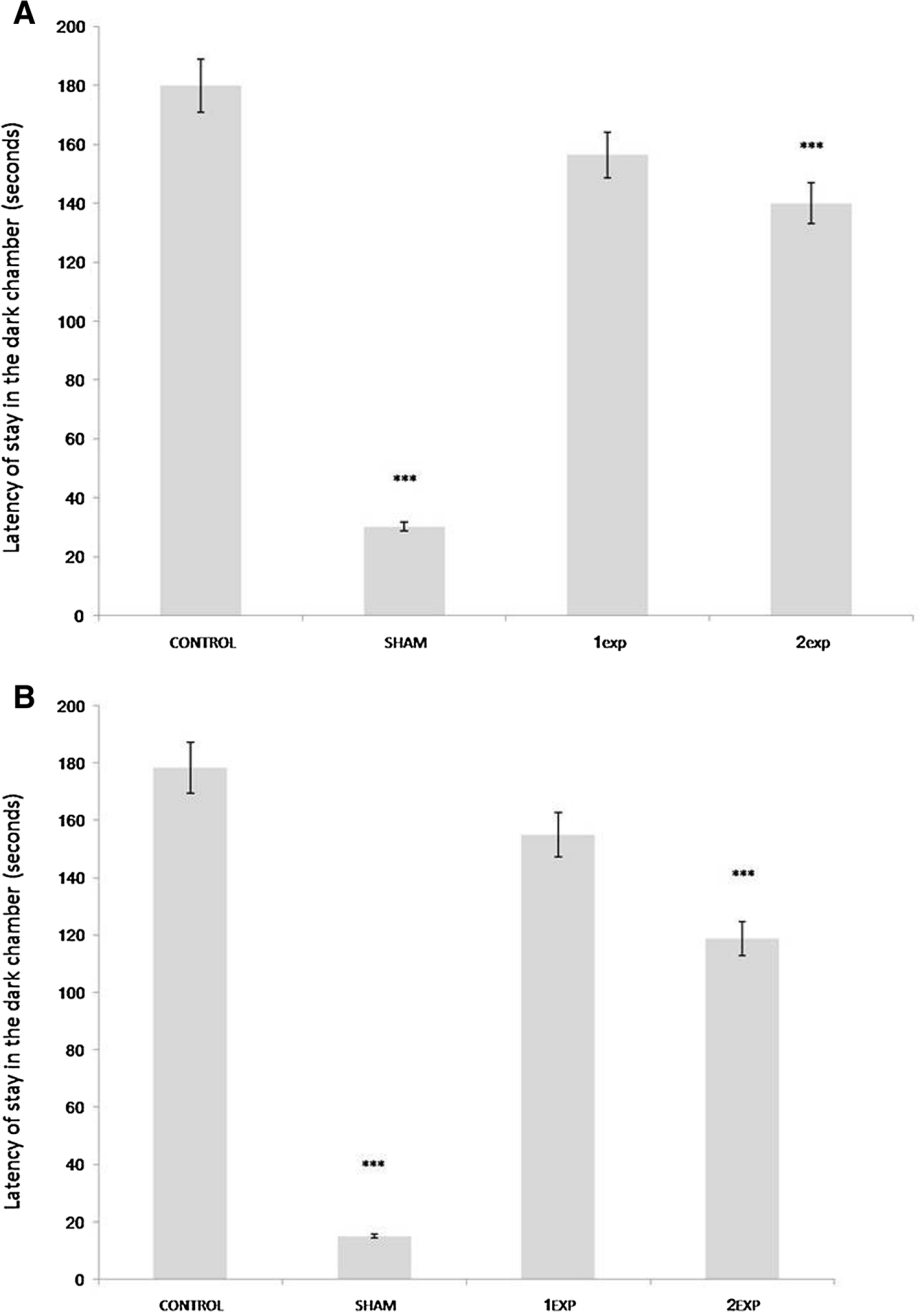
**The mean latency to enter the dark chamber on the training (A) and test day (B).** *** is indicative of (P  0.001). 1Exp group was treated with 70 mg/kg Silymarinan and 2Exp group had received 140mg/kg Silymarin.

Both experimental groups showed significantly longer step-through latency compared to control group (p < 0.05) both in the training (Figure 1A) and test (Figure 1B) days. However, it is interesting to note that the 2Exp group, which had received a higher dose of silymarin showed slightly lower results in comparison with the 1Exp group. The 1Exp group has no significant difference in the step-through latency compared with the control group.

### Histological studies of brain tissue

**Figure 2:**
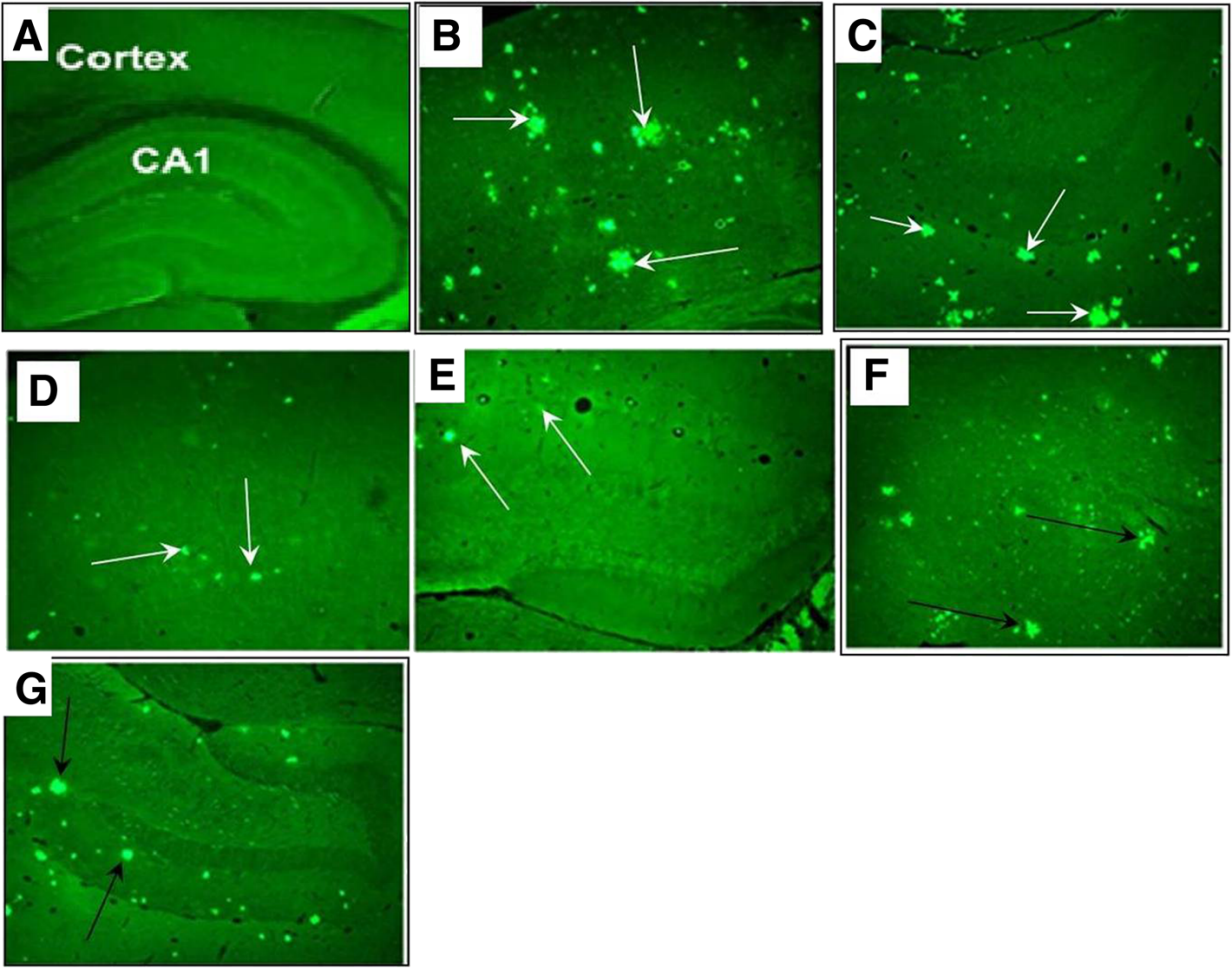
**Brain slices from the frontal cortex &hippocampus with a magnification of 40. (A)**, control group, **(B,C)**, sham group: arrows indicate positions where abeta plaques accumulation has occured, **(D,E)** 1Ex group which was treated with 70 mg/kg silymarin and **(F,G)** 2Ex group which was treated with 140mg/kg silymarin. **B,D,** and **E** correspond to the cortex whereas **C,E,** and **G** correspond to the hippocampus.

ThT staining was used to show the formation of plaques in the abeta-treated brain tissues. Usually, amyloid plaques could be detected in different areas of the brain, including the hippocampus and cortex (for an example of whole brain sections showing plaques distribution see Figure three A,B in reference # [19]). In the present study, both these areas were observed separately with regard to plaques concentration. The normal hippocampus and cortex tissues are showed in Figure 2A. Upon injection of abeta, plaques are formed in both tissues and observed as lighter areas (Figure 2B and C showing the cortex and hippocampus respectively). Treatment with silymarin is effective in diminishing the plaques amount, but here too, administration of 70 mg/Kg of the compound (Figure 2D and E) has a better effect than the higher used dose of 140 mg/Kg (Figure 2F and G).

### Amyloid precursor protein (APP) expression

**Figure 3:**
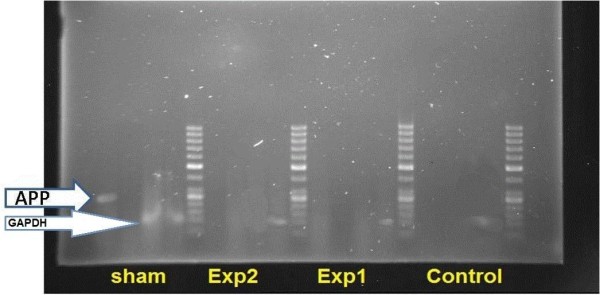
**RT-PCR results comparing gene expression of control, sham and experimental groups.** The locations of the housekeeping gene and APP are indicated by arrows.

Results of electrophoresis are shown in Figure 3. When the housekeeping gene GAPDH is seen in all samples, APP expression is detected in the sham group, which had only received the compound solvent, and is absent in both experimental groups.

## Discussion

From the different theories that try to explain AD pathology, two have been more focused on: the cholinergic theory, which points to the decrease of acetyl choline as the main cause of the disease [20,21] and the amyloid hypothesis, which considers the aggregation of different derivatives of APP as having an important role in the pathophysiology of AD [22]. Therapeutic approaches may include the design of acetyl cholinesterase inhibitors [23] as well as efforts toward finding Aβ aggregation blockers [24], or compounds that would inhibit secretases, i.e. enzymes which cleave APP to Aβ peptides of various length [25]. Some researchers have also tried to employ two or more therapeutic approaches simultaneously, or to find molecules that could affect more than one therapeutic target [26].

Abeta deposits are directly associated with AD. Various isoforms of abeta are derived from the precursor APP, including Aβ25–35 which is one of the most toxic species detected in the brain of AD patients [27,28]. At any rate, administration of abeta variants has led to cognitive impairement [9,29,30] which has also been observed in the present study. The hippocampus is known to be the part of the brain which is first affected by AD [31] and the result of this damage shows itself by cognitive impairement [32] that has been assessed here by the shuttle-box test.

Silymarin, the extract of *Silybum marianum* that contains flavonolignans, has been shown to possess multiple therapeutic properties, including protective effect against nerve damage and brain aging [33,34]. This extract may act via its antioxidant which could protect cells from oxidative stress damage [33], as it has been shown that silymarin effect against oxidative stress may be the result of an increase in reduced glutathione, ascorbic acid and superoxide dismutase levels [35]. Silymarin had also a neuroprotective effect in diabetic mice brains [36], and in mice treated with the brain damaging drug metamphetamine [37]. Previous studies have also reported the beneficial effect of silymarin on Aβ plaques formation, and the effect was suggested to be related to microglial inflammation reduction and direct effect on Aβ accumulation by blocking its aggregation in an Alzheimer’s disease model of transgenic mice [19].

As a matter of fact, silibinin has the ability to inhibit the amyloid structure formation of various model or pathogenic proteins *in vitro*, including Aβ_1-42_[38] human islet amyloid polypeptide [39], and human insulin and albumin [40]*.* This may be suggestive of a generic anti-amyloidogenic effect for this compound, as it has been suggested/observed for various other aromatic/polyphenolic molecules [41,42]. It should be pointed out that in the present study, what has been observed is a disaggregative effect of silymarin on Aβ plaques, since the decrease of plaques was observed *after* they had been formed. This is an important fact, which is directly suggestive of a potential therapeutic effect for the compounds that are able to act on existing fibrils. The current view in the search for AD therapeutic compounds is preferably directed toward compounds that could preserve the native conformation of peptides that may form amyloid structure [43] or to focus on compounds that inhibit the formation of intermediate structures in the course of amyloid formation [44,45]. However, it could make sense to include the compounds that have destabilizing effect on pre-formed fibrils into the spectra of potential AD therapies.

Flavonoids are able to cross the blood brain barrier [46], a fact that should also be true about silymarin, given its established neuroprotective effect [47]. Furthermore, this extract presents few side effects and drug interactions [48] which makes it a quite interesting potential drug for AD. Silymarin is already used as adjuvant therapy in liver insufficiency [49], which is another positive point for a potential drug candidate.

The beneficial effect of silymarin on the short term memory of rats is thus related with less abeta plaques, but also, as demonstrated in the present study, with a negative regulatory effect on the APP gene expression. Increased expression of APP has been detected in the ageing brain [50], and specifically related to AD pathophysiology [51]. Furthermore, the overexpression of APP that is present in the established transgenic mouse model of AD is definitely linked to the pathological characteristics of the model [52].

As far as we know, this should be a first report about the extract effect on APP, which is expanding our knowledge about the mechanism of action of silibinin in attenuating AD symptoms.

## Competing interests

The authors declare that they have no competing interest.

## Authors’ contributions

PY has designed and supervised the project, analyzed the data and advised on writing the paper. This report is part of the results of KA MSc thesis project who performed experiments and wrote the first draft of the manuscript. MD has been an advisor to the thesis, supervised studies and analyzed data. A.E-H. advised on the project, analyzed data, and finalized the manuscript. All authors read and approved the final manuscript.
